# Enhancing NK Cell Activity in Colorectal Cancer with an Fc-Optimized Antibody Targeting CD276 (B7-H3)

**DOI:** 10.2147/ITT.S533342

**Published:** 2026-02-10

**Authors:** Sylwia A Stefańczyk, Xenija Kaiser, Ilona Hagelstein, Martina S Lutz, Samuel J Holzmayer, Latifa Zekri, Melanie Märklin, Susanne Jung

**Affiliations:** 1 Clinical Collaboration Unit Translational Immunology, Department of Internal Medicine, University Hospital of Tübingen, Tübingen, Germany; 2 German Cancer Consortium (DKTK), Partner Site Tübingen, Partnership Between DKFZ and University Hospital Tübingen, Tübingen, Germany; 3 Cluster of Excellence iFIT (EXC 2180) Image-Guided and Functionally Instructed Tumor Therapies, University of Tübingen, Tübingen, Germany; 4 Department of Peptide-Based Immunotherapy, Institute of Immunology, University and University Hospital Tübingen, Tübingen, Germany

**Keywords:** B7-H3, Fc engineering, colon cancer, bench to bedside, immunotherapy, ADCC

## Abstract

**Background & Aims:**

Colorectal cancer (CRC) represents a major global health burden due to its high incidence and mortality, particularly in the advanced stages, with limited treatment options. While therapies that block the PD-1/PD-L1 pathway have demonstrated clinical benefit, their success in CRC is largely confined to patients with high microsatellite instability (MSI-H) tumors. Innovative immunotherapeutic solutions are critically needed for the majority of CRC cases. CD276 (B7-H3), a B7 family immune checkpoint molecule, is overexpressed in many cancers including CRC. Significant CD276 upregulation in CRC cell lines compared to benign tissues has been previously demonstrated. In this study, the potential of an Fc-optimized anti-CD276 monoclonal antibody to enhance natural killer (NK) cell activity in CRC was evaluated.

**Methods:**

An Fc-optimized monoclonal antibody, 8H8_SDIE, was developed to enhance NK cell activity by increasing CD16 binding. In vitro experiments were conducted using human CRC cell lines and peripheral blood mononuclear cells (PBMCs) from healthy adult donors to evaluate the binding specificity of 8H8_SDIE to CD276. NK cell activation was assessed by measuring the upregulation of activation markers (CD69, CD25, and CD107a) and secretion of cytotoxic mediators (IFNγ, granzyme B, and perforin). Cytotoxicity assays were performed to determine NK cell-mediated tumor cell lysis.

**Results:**

8H8_SDIE specifically bound to CD276-positive CRC cells and significantly enhanced NK cell activation. These effects included increased levels of activation and cytotoxic mediators. In cytotoxicity assays, 8H8_SDIE demonstrated potent NK cell-mediated lysis of CRC cells.

**Conclusion:**

The Fc-optimized anti-CD276 antibody 8H8_SDIE effectively enhanced NK cell reactivity against CD276-positive CRC cells and induced tumor cell lysis in vitro. These findings suggest that 8H8_SDIE holds potential as a novel immunotherapeutic candidate for CRC, particularly for patients with microsatellite-stable disease, and warrants further evaluation in advanced preclinical and future clinical studies.

## Plain Language Summary

Colorectal cancer (CRC) ranks among the most frequently diagnosed malignancies and remains a major contributor to cancer-related mortality on a global scale. Many patients with advanced CRC do not respond well to current treatments, and only a small number benefit from existing immunotherapies. This highlights the urgency of developing alternative treatments that could benefit a broader range of patients.

In this study, we focused on a protein called CD276, which is often found in large amounts in cancer cells, including colorectal cancer cells. We created a specially engineered antibody (a type of immune molecule that helps the body recognize and attack threats) targeting CD276. This antibody, named 8H8_SDIE, was optimized to stimulate natural killer (NK) cells - a type of immune cell capable of eliminating tumor cells. We tested the antibody in the laboratory on colorectal cancer cells. The antibody successfully bound to cancer cells and strongly activated NK cells. After activation, NK cells released cytotoxic molecules which induced cancer cell death.

These results suggest that 8H8_SDIE could be a promising new immunotherapy for colorectal cancer. Although more studies are needed before this can be used to treat patients, this study demonstrates a potential approach to improving treatment options for people with this challenging disease.

## Introduction

Colorectal cancer (CRC) is currently the third most frequently occurring cancer worldwide, with increasing diagnosis rates observed particularly among younger individuals. Notably, CRC ranks second in terms of cancer mortality, surpassed by lung cancer.^1^ The considerable mortality of CRC is primarily driven by the poor prognosis in metastatic cases, where the 5-year survival rate is approximately 20%,^2^ and nearly 50% of patients are diagnosed with either primary or secondary metastatic disease.^3^ In the metastatic setting, treatment continues to rely primarily on chemotherapy, often supplemented with monoclonal antibodies (mAbs) that inhibit the epidermal growth factor receptor (EGFR) and vascular endothelial growth factor (VEGFR) pathways;^4–6^ however, these approaches remain non-curative. Immune checkpoint inhibitors (ICIs) have shown success in other cancers, but their efficacy in CRC is largely confined to high microsatellite instability (MSI-H) tumors.^7–10^ Microsatellite-stable (MSS) CRC, which represents the majority of CRC cases, is characterized by low tumor mutational burden and an immunosuppressive tumor microenvironment.^11–13^ These features limit the efficacy of T cell-mediated therapies such as ICIs.^14^ In contrast, natural killer (NK) cells can mediate cytotoxic responses independently of tumor mutational load or antigen presentation, making them promising effectors for treating MSS CRC.^15^,^16^ The absence of similarly effective immunotherapies for the broader CRC patient population, coupled with the dismal prognosis in metastatic disease, underscores the urgency of developing novel treatment strategies.

mAbs have long been an integral component in the management of CRC, with EGFR-targeting agents such as cetuximab or panitumumab applied to impede tumor progression^17^,^18^ and VEGFR-directed antibodies like bevacizumab used to suppress tumor-driven angiogenesis.^19^ While the clinical implementation of these agents has contributed to therapeutic advances, their overall efficacy remains limited, and they are not curative. Efforts to develop antibodies tailored to tumor-specific targets are^20^ and have shown encouraging early-stage results. However, such agents are not yet part of routine clinical use and continue to undergo optimization, for instance, through enhancement of their capacity to trigger antibody-dependent cellular cytotoxicity (ADCC).

ADCC represents a central effector mechanism triggered by mAb therapy, with NK cells serving as the main effector population involved.^21^,^22^ To amplify ADCC responses, the Fc-domain of therapeutic mAb can be structurally modified, either via glycoengineering, as exemplified by the FDA-approved CD20-targeting mAb obinutuzumab used in B cell malignancies, or through the introduction of specific amino acid mutations such as S239D/I332E (SDIE).^23^ These modifications can enhance Fc-binding affinity for Fcγ receptors (FcγR), typically exerting a more pronounced activating effect on FcγRIIIa/CD16a than on the inhibitory FcγRIIb/CD32b.^24^ Nevertheless, expanding the clinical utility of such engineered antibodies to other malignancies requires the identification of suitable tumor-associated targets - antigens that are abundantly expressed on cancer cells but minimally present in healthy tissue.

In this context, CD276 (B7-H3), a member of the B7 immune checkpoint family, has recently attracted interest due to its predominant expression in tumor tissue and its association with the neovasculature and microenvironment, as seen in CRC.^25^,^26^ While it is also detectable at lower levels on certain immune cell subsets, such as antigen-presenting cells and monocytes,^27^ its restricted expression in healthy tissues and consistent overexpression in malignancies support its candidacy as a promising therapeutic target. CD276 is believed to contribute to tumor immune evasion by inhibiting both T and NK cell responses, although its immunomodulatory functions are still being elucidated. Elevated expression of CD276 has been linked to unfavorable clinical outcomes across multiple cancer types.^28–31^ Consequently, CD276 is increasingly recognized as a viable candidate for next-generation therapeutic interventions.^32^,^33^

Our group has previously investigated the Fc-engineered CD276 antibody 8H8_SDIE in various tumor models, including acute myeloid leukemia (AML),^31^ non-small cell lung cancer (NSCLC),^34^ sarcoma,^33^ and others,^35^,^36^ where it showed robust NK cell activation and target cell lysis. Building on these findings, we now investigate whether this immunotherapeutic approach is also applicable to CRC. Given the distinct immune landscape and poor ICI responsiveness of MSS CRC, we sought to explore the therapeutic potential of NK cell-mediated, Fc-enhanced targeting of CD276 in this context.

Accordingly, we characterized CD276 expression in CRC cell lines and assessed the ability of 8H8_SDIE, a novel Fc-engineered CD276 mAb, to activate NK cells and enhance their cytotoxic response against CRC.

## Materials and Methods

### Peripheral Blood Mononuclear Cells and Cell Lines

CRC cell lines, including CaCo-2, Colo-205, HCT-116, HT-29, and SW620, were obtained from the German Collection of Microorganisms and Cell Cultures (Braunschweig, Germany) and the American Type Culture Collection (Manassas, VA, USA). All cell cultures were routinely screened for mycoplasma contamination on a quarterly basis. Authentication of cell lines was confirmed by flow cytometric immunophenotyping, following supplier-provided guidelines.

Peripheral blood mononuclear cells (PBMCs) were collected from healthy adult donors of varying age and sex, randomly assigned to individual experiments. Isolation was performed via Ficoll-based density gradient centrifugation (PAN-Biotech, Aidenbach, Germany, Cat#P04-601,000), and PBMCs were cryopreserved in liquid nitrogen until needed. Before use in functional assays, cells were thawed and incubated at 37°C for 24 h in RPMI 1640 medium enriched with GlutaMAX (Thermo Fisher Scientific, Cat#72400-054), 10% heat-inactivated fetal calf serum (PAN-Biotech, Aidenbach, Germany, Cat#FBS-HI-12A), and antibiotics (penicillin 100 U/mL and streptomycin 100 μg/mL; Sigma-Aldrich, St. Louis, USA, Cat#P06-07100). Healthy donor samples were randomly selected without predefined criteria to reflect biological variability and avoid selection bias. Key experiments were conducted with ≥3 independent donors using standardized protocols across different days to minimize batch effects.

PBMCs were used instead of purified NK cells to better replicate the physiological cellular context and to preserve accessory interactions that influence NK cell activation and cytotoxicity. All donors provided written informed consent. The study was approved by the Ethics Committee of the University of Tübingen and conducted in compliance with the Declaration of Helsinki.

### Antibody Production and Purification

A mouse-derived anti-human CD276 mAb (clone 8H8), was previously generated and chimerized by fusing its variable regions to the constant domains of human immunoglobulin G1/κ (IgG1/κ) to yield a human-compatible antibody format.^31^ Fc-optimization was performed by introducing the S239D/I332E (SDIE) amino acid substitutions into the Fc-region, which are known to increase binding affinity for FcγRIIIa/CD16a and enhance NK cell-mediated ADCC. The generation and engineering strategy was originally established and validated in the context of FLT3-targeting antibodies, as described by Hofmann et al (2012).^37^ For antibody production, heavy and light chain plasmids were prepared using the EndoFree Plasmid Maxi Kit (Qiagen, Hilden, Germany) and transfected into ExpiCHO cells (Gibco, Carlsbad, CA) according to the manufacturer’s instructions. Antibodies were purified from cell culture supernatants via protein A affinity chromatography (GE Healthcare), followed by size-exclusion chromatography (HiLoad 16/60 Superdex 200, GE Healthcare) to ensure monomeric antibody preparation. Purity and integrity were verified by analytical SEC (Superdex 200 Increase 10/300 GL, GE Healthcare) and 4–12% SDS-PAGE (Invitrogen, Carlsbad, CA), confirming expected molecular weight and absence of degradation products. The isotype control antibody (MOPC_SDIE) was generated analogously using the murine MOPC-21 clone chimerized with human IgG1/κ constant regions and modified with SDIE mutations to match the Fc-profile of 8H8_SDIE.

### Flow Cytometry

Prior to flow cytometric analysis, cells were preincubated with human or mouse IgG (Merck KGaA, Darmstadt, Germany, Cat#I4506; Southern Biontech, Birmingham, AL, USA, Cat#0107-01 respectively) to reduce nonspecific antibody binding. Subsequently, cells were stained with mouse anti-human CD276-PE/Cy7 (clone MIH42, BioLegend, San Diego, CA, USA, Cat# 351008), 8H8_SDIE, or corresponding isotype controls (Cat #557872; BD Pharmingen, San Diego, CA, USA). Secondary detection was carried out using goat anti-mouse PE (DAKO, Glostrup, Denmark, Cat# R0480) or goat anti-human PE (Jackson ImmunoResearch, West Grove, PA, USA, Cat# 109–116-097) antibody. Fluorescent antibodies CD3-APC (clone SK7, BD Pharmingen, Cat# 555335) and CD56-PE/Cy7 (clone HCD56, BioLegend, Cat# 318318) were employed to identify NK cells. Intracellular cytokines IFNγ and TNF were detected following incubation with GolgiStop and GolgiPlug reagents (BD Biosciences, Heidelberg, Germany, Cat#554724, 55029, respectively). After CD56 staining, cells were fixed and permeabilized using a commercial permeabilization buffer system (Fixation/Permeabilization Solution Kit, BD Biosciences, Heidelberg, Germany, Cat#51-2090KZ). Intracellular staining involved mouse anti-human IFNγ-BV421 (clone B27, Cat# 506538) and mouse anti-human TNF-BV785 (clone Mab11, Cat#502948; both from BioLegend). For cytotoxicity assays, CRC cells were labeled with CellTrace™ Violet proliferation dye (2.5 mM; Thermo Fisher Scientific, Cat# C34557) and co-cultured with PBMCs from healthy volunteers, in the presence or absence of the antibodies (1 µg/mL). To standardize acquisition volume, silicone beads (Merck KGaA, Cat#LB30-2ML) were added to all tubes. Non-viable cells were excluded by viability dye staining with either 7-AAD (BioLegend, Cat# 420404) or LIVE/DEAD™ Fixable Aqua (Cat# L34966; Thermo Fisher Scientific). Flow cytometry was conducted using FACS CANTO II or FACS Fortessa instruments (BD Biosciences), and data were processed using FlowJo v10 software (FlowJo LLC, Ashland, OR, USA). Surface marker expression was calculated as mean fluorescence intensity (MFI) and represents the absolute fluorescence signal of antigen-stained versus isotype control-stained populations. CD276 expression was initially validated using a commercial antibody (BioLegend) to confirm target presence in CRC cell lines. This was followed by functional and binding analysis using 8H8_SDIE. Binding results were interpreted qualitatively and not used to directly compare binding affinities.

Flow cytometry gating strategies for NK cell subsets and activation markers (CD107a, CD25, CD69) are provided in Supplementary Figure S1. For each sample, a minimum of 100,000 events were acquired after gating on lymphocytes. Unstained, single-stained, and fluorescence-minus-one (FMO) controls were included to ensure accurate gating.

### Analysis of NK Cell Function: Activation, Degranulation, and Cytokine Secretion

To evaluate NK cell activation, PBMCs derived from healthy donors were co-cultured with CRC cells at an effector-to-target (E:T) ratio of 2.5:1, using 500,000 PBMCs and 200,000 tumor cells per condition. Treatments were applied at a concentration of 1 µg/mL during co-incubation. To assess NK cell degranulation, GolgiPlug and GolgiStop (BD Biosciences) were added to the culture medium, and after 4 hours, cells were harvested, characterized as CD3^−^ CD56^+^, and stained with CD107a-PE (clone H4A3, BD Pharmingen, Cat# 555801), and a viability dye (Fixable Aqua), followed by flow cytometry. NK cell activation was further evaluated by staining with CD69-PE (clone FN50, BD Pharmingen, Cat# 555531) and CD25-PE (clone BC96, BioLegend, Cat# 302606) after 24 and 72 h. Cytokine production was determined in cell-free supernatants collected after 24 h of culture. The concentrations of granzyme A, granzyme B, perforin, granulysin, TNF, IL-2, IFNγ, and IL-10 were quantified using the Legendplex multiplex bead-based assay (BioLegend, Cat# 741186) following the manufacturer’s protocol.

### Assessment of NK Cell Cytotoxicity

The ability of NK cells within PBMCs from healthy individuals to lyse CRC cell lines was investigated by co-incubation in the presence or absence of 8H8_SDIE or MOPC_SDIE (1 µg/mL), using the DELFIA Cell Cytotoxicity Assay (Perkin Elmer, Waltham, MA, USA, Cat# C136-100; C135-100). After a 2-hour incubation, cytotoxicity was quantified according to the supplier’s instructions. Specific lysis was calculated based on the following equation:

Specific lysis (%) = 100 × (experimental release - spontaneous release)/(maximum release - spontaneous release)

To monitor cytotoxicity dynamically, the xCELLigence RTCA system (Roche Applied Science, Penzberg, Germany) was employed. CRC cell lines were seeded into 96-well electronic plates and incubated for 24 h to allow adherence. Subsequently, PBMCs from healthy donors were added to the wells and co-cultured with tumor cells at an E:T ratio of 40:1, either in the presence or absence of 1 µg/mL 8H8_SDIE and its control antibody. Real-time impedance-based readouts of cytolytic activity were collected every 15 min for up to 150 h, generating detailed cytotoxicity kinetics.

### Transcriptomic Data Mining

CD276 transcript levels were evaluated using public RNA-seq data retrieved from the UCSC Xena platform (dataset: TCGA TARGET GTEx: Gene expression RNAseq: TOIL RSEM TPM; host: toil.xenahubs.net). Specifically, TCGA COADREAD (colon and rectum adenocarcinoma) samples were compared with GTEx colon normal tissue.

### Statistical Analysis

Unless stated otherwise, data are presented as mean ± standard error of the mean (SEM). Appropriate statistical methods were selected based on the distribution and design of each experiment, including Student’s *t*-test, one-way ANOVA, Mann–Whitney *U*-test, or Log rank test as applicable. All analyses were carried out using GraphPad Prism version 10.1.1. A threshold of p < 0.05, was considered statistically significant, with significance levels annotated as *p < 0.05, **p < 0.01, and ***p < 0.001. Significance indicators (p < 0.05) were only applied to comparisons involving adequately powered sample sizes (n ≥ 3), while non-significant results were reported without annotation.

## Results

### CD276 Surface Expression on CRC Cell Lines

**Figure 1:**
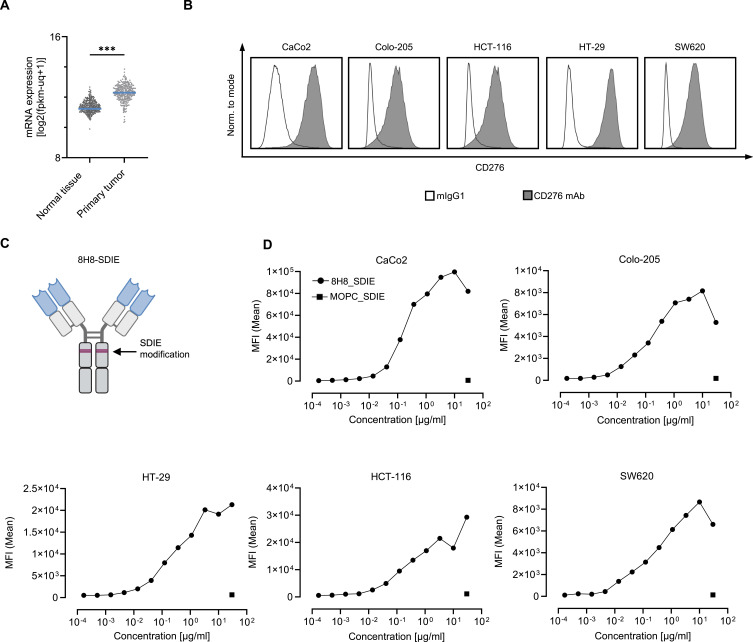
Characterization of CD276 expression in CRC cell lines. (**A**) Analysis of CD276 mRNA expression in normal colon tissues (n = 308) versus primary adenocarcinoma tumor (n = 288) using publicly available TCGA, TARGET, and GTEx datasets through the UCSC Xena platform.^38^ (**B**) Flow cytometric evaluation of CD276 surface expression on CaCo2, Colo-205, HCT-116, HT-29, and SW620 cell lines. Cells were stained with the CD276-PE/Cy7 antibody or isotype control, and representative histograms from three independent experiments are shown. Norm.- normalized. (**C**) Schematic representation of the humanized CD276 monoclonal antibody (8H8_SDIE) engineered with an Fc-region optimized for enhanced binding to CD16 on NK cells. (**D**) Binding activity of 8H8_SDIE was assessed by flow cytometry on CRC cell lines by dose titration. ***p < 0.001.

To examine CD276 transcript levels, data retrieved from the TGCA, TARGET, and GTEx databases were analyzed via the Xena platform,^38^ comparing normal colon tissues (n = 308) with primary adenocarcinoma specimens (n = 288). The results demonstrated significantly elevated CD276 mRNA levels in adenocarcinoma relative to normal tissues (Figure 1A). Next, CD276 surface expression was assessed in several CRC cell lines, including: CaCo2, Colo-205, HCT-116, HT-29, and SW620. Flow cytometry indicated high CD276 protein levels in all tested cell lines (Figure 1B).

Figure 1C provides a diagram of the Fc-engineered CD276 antibody, 8H8_SDIE. To test the binding of the antibody to its target, flow cytometry was used to evaluate the binding of 8H8_SDIE to CRC cell lines across a range of concentrations. Binding intensity, measured as MFI, increased up to 1 μg/mL and reached saturation between 1 and 10 μg/mL across all tested CRC cell lines, (Figure 1D). These results highlight both the elevated expression of CD276 in CRC and stable target engagement of 8H8_SDIE in CRC models and support its use in functional assays.

### Activation of NK Cells by 8H8_SDIE in CD276-Positive CRC Co-Culture Models

**Figure 2:**
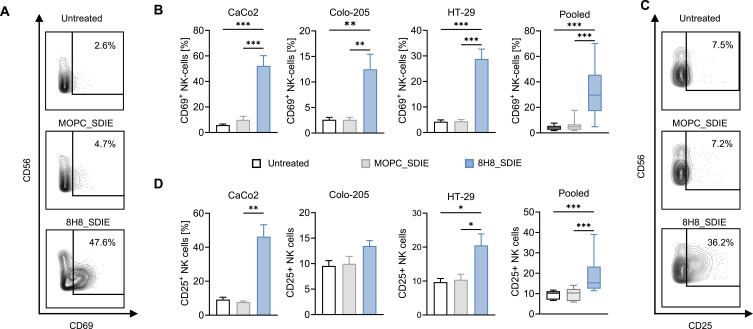
Activation of NK cells by Fc-optimized CD276 antibody. PBMCs from healthy donors (n = 4) were co-cultured with CRC cell lines at an E:T ratio of 2.5:1 in the presence or absence of the 8H8_SDIE antibody or isotype control MOPC_SDIE (both at 1 µg/mL). NK cell activation was assessed by flow cytometry, measuring CD69 expression after 24 h and CD25 expression after 72 h. (**A**) Representative flow cytometry plots of CD69 expression in NK cells co-cultured with CaCo2 cells at 24 h. (**B**) Individual (left) and pooled (right) flow cytometry data for CD69 expression in NK cells co-cultured with the indicated CRC cell lines. (**C**) Representative flow cytometric plots showing CD25 expression in NK cells after 72 h of coculture with HT-29 cells. (**D**) Individual (left) and pooled (right) flow cytometry data for CD25 expression in NK cells co-cultured with CRC cell lines. *p < 0.05; **p < 0.01; ***p < 0.001.

To assess whether 8H8_SDIE can enhance NK cell activity against CRC targets, co-culture assays were conducted using PBMCs derived from four healthy donors and three CRC cell lines (CaCo2, Colo-205, and HT-29). NK cell activation was evaluated under treatment with either 8H8_SDIE or MOPC_SDIE. After 24 h of incubation, flow cytometric results showed a marked increase in CD69 expression, an early marker of NK cell activation, in cells treated with 8H8_SDIE compared to MOPC_SDIE (Figure 2A and B). Prolonged co-culture for 72 h further demonstrated a notable upregulation of CD25, which is linked to sustained NK cell stimulation and proliferative capacity, again specifically in 8H8_SDIE-treated NK cells. MOPC_SDIE did not elicit such effects (Figure 2C and D). Collectively, these findings demonstrate the ability of 8H8_SDIE to promote NK cell activation through its interaction with CD276 on CRC cells.

### Induction of NK Cell Reactivity and Cytotoxicity Against CD276-Positive CRC Cell Lines

**Figure 3:**
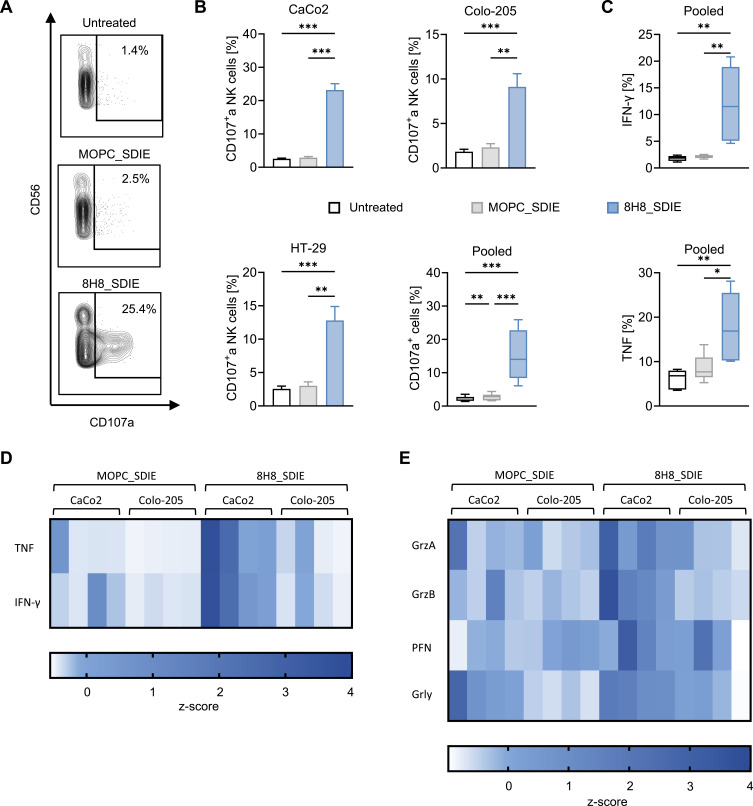
Enhancement of NK Cell degranulation and cytokine secretion by Fc-optimized CD276 antibody. PBMCs from healthy donors were co-cultured with CRC cell lines at an E:T ratio of 2.5:1 in the presence or absence of the Fc-optimized 8H8_SDIE antibody or isotype control MOPC_SDIE (both at 1 µg/mL). NK cell degranulation and cytokine responses were evaluated as follows: (**A**) Representative flow cytometric plots showing CD107a expression in NK cells co-cultured with CaCo2 cells after 4 h. (**B**) Individual and pooled flow cytometric data showing CD107a expression in NK cells co-cultured with CRC cell lines and PBMCs from healthy donors (n = 4). (**C**) Intracellular expression of IFNγ (upper panel) and TNF (lower panel) in NK cells identified by CD3^−^CD56^+^ counterstaining and analyzed using flow cytometry after 4 h of co-culture. (D+E) Supernatants were analyzed after 24 h for (**D**) immunoregulatory cytokines TNF and IFNγ, and (**E**) effector molecules, such as granzyme A, granzyme B, perforin, and granulysin, as assessed by the Legendplex multiplex assay. Heatmaps showing individual results for CRC cell lines and PBMCs donors (n = 4). *p < 0.05; **p < 0.01; ***p < 0.001.

To investigate whether 8H8_SDIE augments NK cell reactivity toward CRC cell lines, co-culture assays were conducted to evaluate NK cell degranulation and cytokine output. NK cell degranulation, an early indicator of cytotoxic activity, was assessed by measuring the CD107a expression after 4 h. NK cells co-cultured with CRC cells in the presence of 8H8_SDIE exhibited a substantial increase in CD107a expression relative to those threated with the control antibody (Figure 3A and B).

To explore the broader immunostimulatory potential of 8H8_SDIE, intracellular levels of IFNγ and TNF were assessed by flow cytometry. A clear enhancement of these cytokines was noted in NK cells exposed to 8H8_SDIE when compared to MOPC_SDIE-treated cells (Figure 3C). In line with these results, multiplex assays conducted on supernatants harvested after 24 h revealed elevated concentrations of IFNγ, TNF, and cytotoxic effector proteins such as granulysin, granzyme A, granzyme B, and perforin (Figure 3D and E). In summary, these data highlight the immunopotentiating effects of 8H8_SDIE, which drive both cytokine-mediated signaling and direct NK cell cytotoxicity toward CRC targets.

### Induction of NK Cell Lysis Against CD276-Positive CRC Cells

**Figure 4:**
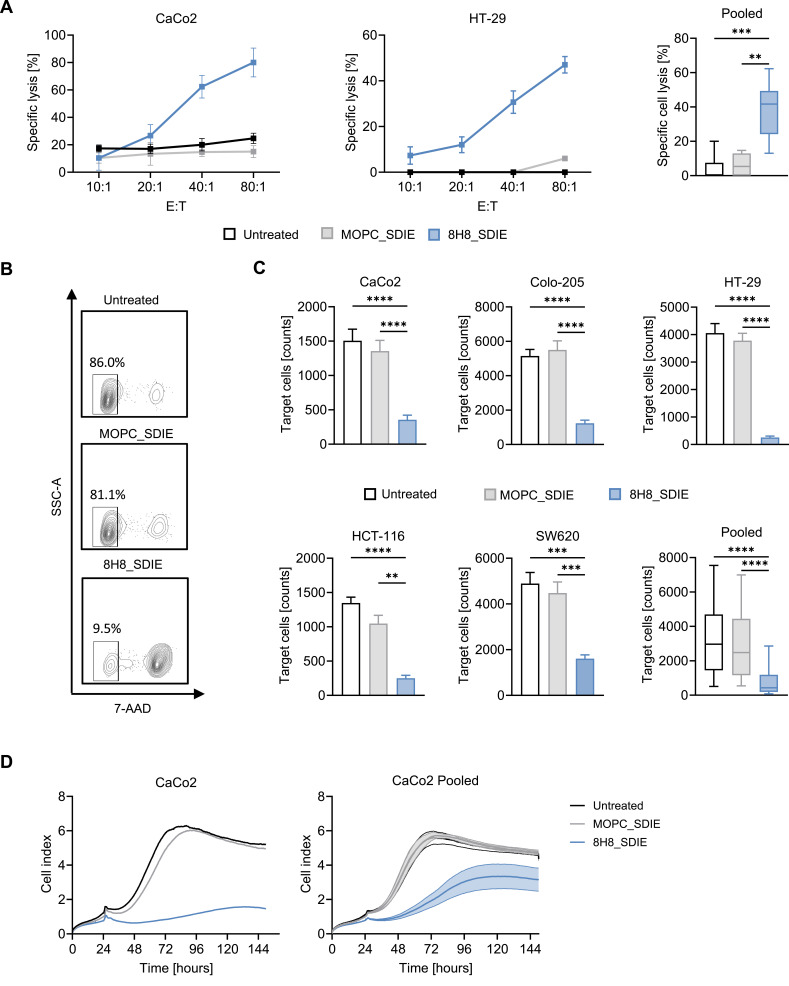
Enhanced targeted cell lysis of CRC Cell lines by Fc-optimized 8H8_SDIE antibody. The cytotoxic effects of Fc-optimized 8H8_SDIE were evaluated in coculture models of PBMCs from healthy donors and CRC cell lines under different conditions. (**A**) Europium-based cytotoxicity assays were performed after 2 h of coculture at different E:T ratios. The left panels show representative results for CaCo2 and HT-29 cells, whereas the right panel shows pooled data for CaCo2, Colo-205, and HT-29 cells at an E:T ratio of 40:1 with multiple PBMC donors (n = 2–4). (**B**) Exemplary plots showing flow cytometry analysis after 24 h of co-culture with living (7-AAD^−^) CaCo2 cells at an E:T ratio of 20:1. (**C**) Individual and pooled flow cytometry results after 24 h of co-culture for lysis of five CaCo2, Colo-205, HT-29, HCT-116, and SW620 cells with different PBMC donors (n = 3–4). (**D**) Long-term cytotoxicity was assessed using an impedance-based xCELLigence real-time cell analysis system over 150 h of coculture at an E:T ratio of 40:1. The left panel shows representative results for a single PBMC donor co-cultured with CaCo2 cells, and the right panel shows pooled data from multiple donors (n = 4). **p < 0.01; ***p < 0.001; ****p < 0.0001.

To evaluate whether the increase in NK cell reactivity and cytotoxic function triggered by 8H8_SDIE translates into efficient lysis of CRC cells, PBMCs from healthy donors were co-cultured with or without 8H8_SDIE or MOPC_SDIE. The short-term cytotoxicity was determined after 2 h of incubation. Significant target cell lysis of CRC cells was observed in the presence of 8H8_SDIE (Figure 4A). To verify these results across an extended timeframe, flow cytometric analysis was performed at 24 hours post co-culture. A marked decline in viable CRC cell numbers was observed in 8H8_SDIE-treated samples as compared to those exposed to MOPC_SDIE (Figure 4B and C). Complementary real-time cytotoxicity profiling using the impedance-based xCELLigence system over a 150-hour period further substantiated the cytolytic capacity of 8H8_SDIE (Figure 4D). Collectively, these data indicate that CRC cell lysis mediated by NK cells is significantly boosted by 8H8_SDIE in both short and long-term assays, underscoring its potential as a viable therapeutic candidate for colorectal cancer.

## Discussion

Over the past decade, there have been significant improvements in CRC treatment. By rolling out early screening strategies and successfully treating early disease manifestations, it has been possible to lower the overall mortality rate of CRC.^39^ However, the group of individuals diagnosed with advanced stage IV metastatic disease has not benefited from this positive trend. Except for patients with MSI-H status or loss of DNA mismatch repair, there have been no significant additions to decades-old treatment regimens consisting of chemotherapy and EGFR- or VEGFR-targeted antibodies. Consequently, the long-term survival rate in this cohort remains low, with only 16% reaching 5-year survival compared to 91% in the early stages.^39^ However, the dramatically increasing number of younger patients^40^ who fail to be diagnosed using classic early detection schemes continues to expand this particular patient cohort,^41^ keeping CRC at an overall second for cancer-related mortality. In light of this urgent clinical need, our study provides a rationale for targeting CD276 in CRC using the Fc-optimized antibody 8H8_SDIE, which robustly activated NK cells and mediated cytotoxicity across multiple CRC cell lines.

Here, we introduce a novel approach that is particularly suitable for this underserved patient population. After successfully demonstrating that CRC cells and their tumor environment and neovasculature predominantly express the target antigen CD276,^26^,^42^ we constructed a mAb against this antigen, which is able to potently activate NK cells, which serve as the key cytotoxic effector subset, with long-lasting and enhanced ADCC. This represents a novel immune-based intervention, potentially benefiting a broader fraction of patients with metastatic CRC. Similar NK cell-mediated anti-tumor effects of 8H8_SDIE have previously been reported in several preclinical models of hematologic and solid malignancies, including acute myeloid leukemia and lung cancer,^31^,^34^ underscoring its translational relevance.

To date, immunotherapy has been restricted to the use of pembrolizumab for the treatment of metastatic CRC with MSI-high instability, accounting for up to 15% of all CRC cases.^43^ This was based on promising data from the KEYNOTE and CheckMate studies, which showed significantly better outcomes with fewer side effects.^44–46^ However, in this particular subset of CRC, mutations in MLH1 or MSH2 cause mismatch repair deficiency (dMMR), which in turn leads to a failure in the cellular detection and correction of spontaneous DNA mutations, culminating in a very high tumor mutation burden. The therapeutic effect of immune checkpoint inhibition (ICI) is based on this specific pathogenic pattern, as shown in previous studies across several tumor entities with dMMR, including CRC.^47^,^48^ However, the vast majority of CRC cases develop via different pathogenetic mechanisms, commonly progressing through the traditional adenoma-to-carcinoma pathway involving APC gene mutations, creating tumors with a low mutational burden. While we did not experimentally determine the mutational status of the CRC cell lines in this study, publicly available genomic datasets and prior literature identify HCT-116 as MSI-high,^49^,^50^ whereas HT-29 and SW620 represent MSS phenotypes.^49^,^51^,^52^ The ability of 8H8_SDIE to activate NK cells and elicit cytotoxicity across this panel suggests that its efficacy may extend across mutational subtypes,^52^ though further work is required to confirm this in clinically annotated samples.

As immunotherapy, especially ICI, continually gains traction in other gastrointestinal tumor entities,^53–57^ increased efforts have been undertaken to make microsatellite stable CRC amenable to ICI. The driving theory behind these efforts is that a combination of more than one (immune)therapeutic agent can potentially evoke an immunogenic response, enabling ICI efficacy.^58^ Combinatorial chemotherapy (NCT03832621, NCT03608046), radiotherapy (NCT03007407, NCT03104439) or even the antidiabetic drug metformin (NCT03800602) have been proposed as possible partners for ICI, but also other immunotherapeutic agents such as vaccines or combinatorial ICI regimens. Most of these studies are still ongoing, but some preliminary results seem to indicate that the attempts to evoke immune responses have not been entirely successful so far.^59^ Given that CD276 is consistently expressed across CRC models and 8H8_SDIE efficiently induces NK cell responses in vitro, this approach may offer a complementary or alternative route to overcome ICI resistance in MSS CRC, where current immunotherapies have shown limited success. Notably, in contrast to other tumor types we have studied, such as AML, NSCLC, ovarian cancer, or sarcomas, CRC models exhibited a more heterogeneous profile of cytokine and effector molecule release in response to 8H8_SDIE, highlighting the relevance of tumor-type–specific immune modulation.

Another immunotherapeutic approach currently being researched and developed for CRC is adoptive cell therapy, particularly T cells engineered to express chimeric antigen receptors (CAR-T cells), which have been explored in multiple early-phase clinical studies and target several different tumor antigens, including, but not limited to, carcinoembryonic antigens (NCT02850536, NCT02416466, etc)., NKG2DL (NCT05248048, NCT04550663, etc)., and EGFR (NCT03542799, NCT03152435). However, promising data on the therapeutic potential of CAR-T cells have been juxtaposed with the several challenges arising from these studies. For example, most of the currently investigated CAR-T cell target antigens are not entirely tumor-exclusive, which causes toxicity and limits therapeutic use. Second, there is insufficient expansion and persistence of CAR-T cells in circulation or within the tumor, and the tumor tissue invasion of CAR-T cells is low.^60^ Because mAbs do not rely on the proliferation of a manufactured cell product, but instead employ the body’s own immune cells, they avoid the particular problem of ongoing proliferation. Because the target antigen of 8H8_SDIE is upregulated in the tumor and tumor environment, including the neovasculature, off-tumor toxicities and side effects are rare, and access to the tumor should be vastly improved. While toxicity issues remain to be addressed in clinical Phase I trials, in a comparison of different immunotherapeutic approaches, mAbs, especially 8H8_SDIE, appear to avoid many of the problems associated with currently explored CAR-T cell approaches. Importantly, in vivo studies using 8H8_SDIE in acute myeloid leukemia (AML) models have previously demonstrated a favorable safety profile, with no evidence of off-tumor toxicities or systemic immune activation.^31^ These findings support the translational potential of this Fc-engineered antibody and warrant further investigation in solid tumors such as CRC.

Notably, 8H8_SDIE is still in a very early stage of development. Although we deliberately used PBMCs from healthy donors to preserve physiological immune context and accessory interactions relevant for ADCC,^61^ future mechanistic studies may benefit from using purified NK cell subsets to dissect effector-intrinsic signaling in greater detail. While these cell line-based and ex vivo assays provide valuable insights, they do not fully represent the complexity of the tumor microenvironment in CRC, which is characterized by multifaceted interactions among malignant epithelial cells, supportive stromal cells, and extracellular matrix components.^62^,^63^ Advanced in vivo models, such as patient-derived xenografts and humanized mice, are essential for replicating human tumor dynamics and evaluating therapeutic efficacy in more clinically relevant settings. Additionally, detailed biodistribution analyses are necessary to establish the safety characteristics of 8H8_SDIE prior to its advancement into Phase I clinical trials. To further support the translational development of 8H8_SDIE specifically in the context of colorectal cancer, future studies could incorporate non-transformed epithelial models or patient-derived colon organoids to assess potential off-tumor effects under more physiological conditions.

With evidence supporting tumor-enriched CD276 expression and our results establishing enhanced lysis of CRC cells mediated by NK cells and triggered by 8H8_SDIE in both short- and long-term functional assays, further studies and trials are warranted given its potential as an effective therapeutic strategy for a hitherto underserved patient collective.

## Conclusions

These results support the further development of 8H8_SDIE as a targeted immunotherapeutic strategy for colorectal cancer. By enhancing NK cell activation through Fc-engineering, 8H8_SDIE improves ADDC, which is the central mechanism underlying its anti-tumor activity. This antibody may provide a much-needed treatment alternative for patients with microsatellite-stable (MSS) CRC, a subgroup that comprises the majority of CRC cases and currently lacks effective immunotherapeutic options. While previous in vivo studies in AML models demonstrated a favorable safety profile, future research should include comprehensive in vivo assessments in solid tumor settings to confirm the safety and biodistribution profile of 8H8_SDIE prior to clinical translation.

## Data Availability

Datasets supporting the conclusions of this study were included in this study. Additional data were provided by the corresponding author upon request.

## Abbreviations

7-AAD, 7-aminoactinomycin D; ADCC, antibody dependent cellular cytotoxicity; CAR, chimeric antigen receptor; CRC, colorectal cancer; ET, effector to target; FACS, Fluorescence-activated cell sorting; FcγR, Fc-gamma receptor; HD, healthy donor; ICI, immune checkpoint inhibition; IFNγ, Interferon-γ; mAb, monoclonal antibody; MFI, mean fluorescence intensity; dMMR, Mismatch Repair deficiency; MSI, microsatellite instability; NK cell, natural killer cell; PBMC, peripheral blood mononuclear cell; PD-1/PD-L1, programmed death- 1/ programmed death-ligand 1; PE, phycoerythrin; SEM, standard error of the mean; SFI, specific fluorescence intensity; TMB, tumor mutation burden; TME, tumor microenvironment.
